# Continue or not to continue? Attitudes towards deprescription among community-dwelling older adults in China

**DOI:** 10.1093/geroni/igab046.3043

**Published:** 2021-12-17

**Authors:** Jie Tan, Li Zhang, Ying Wang, Lihui Chen, Chenkai Wu

**Affiliations:** 1 Duke Kunshan University, Kunshan, Jiangsu, China (People's Republic); 2 Suzhou Municipal Hospital, Kunshan, Jiangsu, China (People's Republic)

## Abstract

Inappropriate prescribing of medications and polypharmacy among older adults could lead to avoidable harms. It is hence vital to stop potentially inappropriate medications in this vulnerable group. An approach coined 'deprescribing' has been used to describe a patient-centerd process of optimizing medication regimens. But patient resistance to discontinuing medication use is a significant barrier to deprescribing. The present study aims to describe attitudes towards deprescribing and to examine individual-based characteristics that might be associated with these attitudes among community-dwelling older adults in China. We conducted a cross-sectional study through in-person interviews using the validated Patients’ Attitudes Towards Deprescribing questionnaire in two communities through the community-based physical examination platform. Participants were 65 years and older and had at least one chronic disease and one regular prescription medication. Of the 1,897 participants in the study, the average age was 74 years and 1,023 (53.9%) were women. The majority had one chronic disease (n=1,364 [71.9%]) and took 1-2 medications (n=1,483 [78.2%]). A total of 947 (50.0%) older adults reported being willing to stop taking one or more of their medicines if their physician said it was possible, and 1204 (63.5%) older adults wanted to stop a medicine been taking for a long time. Chronological age, marital status, number of chronic diseases, and self-rated health status were associated with the attitudes towards deprescribing. This study showed that half of the participants were willing to cease a medication that their physician though was no longer required. Individual-level factors were associated with attitudes towards deprescribing.

